# Supplementary material for: Evaluation of a new implant for Tibial Tuberosity Transposition in dogs: An ex vivo study
Source: PLoS One. 2025 Nov 4;20(11):e0335274. doi: 10.1371/journal.pone.0335274 (PMC12585020; doi:10.1371/journal.pone.0335274)
Supplement: Attachment — Submitted filename: Response to Reviewers.docx [file pone.0335274.s003.docx]

Dear Academic Editor and Reviewers

Re: PONE-D-25-12917 “Evaluation of a New Implant for Tibial Tuberosity Transposition in Dogs: An Ex Vivo Study”

We would like to thank you for considering publishing this article and carrying out a major review. We believe that the referees presented thoughtful and constructive comments on our manuscript, and we are comfortable in addressing each one in this revised version.

Please find below responses to each comment. Changes in the manuscript are highlighted in red in a ‘tracked’ file. The highlighted version and a clean version of the revised manuscript were uploaded to the PLOS One submission site. All authors have read and approved the revised version of the manuscript as submitted.

To clarify the funding sources, Engevet provided partial financial support for the research, specifically covering the manufacturing of the implant. The funders had no role in study design, data collection and analysis, decision to publish, or preparation of the manuscript.

This study was financed in part by the Coordenação de Aperfeiçoamento de Pessoal de Nível Superior - Brasil (CAPES) – Finance Code 001. Author GR received a research fellowship from CAPES, which supported the development of this study. The funders had no role in study design, data collection and analysis, decision to publish, or preparation of the manuscript.

We hope that you find our responses satisfactory. Thank you for your consideration of our manuscript. If you need any additional information, please, do not hesitate to contact us.

Yours sincerely,

The Authors

**Evaluation of a New Implant for Tibial Tuberosity Transposition in Dogs: An Ex Vivo Study**

**Response to Academic Editor**

**The objective describes the desire to evaluate the complications of the proposed TTT technique. However, this static cadaveric study (which does not include a dynamic biomechanical study) has provided as outcomes only the evaluation of the integrity of the tibial tuberosity and the implant immediately after transposition and without any load. Most of the studies cited in discussion and compared with this one (both clinical studies and ex-vivo studies) have evaluated the complications inherent to the integrity of the bone and the implant by providing a load.**

R: We thank the editor for the correction. In response to the comments and suggestions from the editor and reviewers, the manuscript has been duly revised. We agree with the consideration that the present study was static and was limited to assessing the occurrence of complications at the time of tibial tuberosity displacement. Consequently, the study's objective has been readjusted to strictly reflect the outcomes assessable within its scope. We would like to emphasize, however, that the primary objective behind the development of this implant is to demonstrate its intrinsic capacity to perform tibial tuberosity transposition slowly and gradually. This functionality obviates the need for specific surgical instrumentation or auxiliary devices for this purpose, thereby aiming to simplify the surgical procedure and enhance its precision. To this end, the manuscript has been revised not only to clarify the objective but also to adjust the discussions regarding the assessment of complications (in the Materials and Methods, Results, Discussion and Conclusion sections), particularly concerning the comparison with other biomechanical and clinical studies. Below are some excerpts with the modifications made.

Ln28: The samples underwent radiographic evaluation and manual palpation following the application to detect tibial crest fractures. The plate's shape adequately fit the medial surface of the tibia, and the implant was effective in promoting a slow and gradual transposition in canine cadavers without the need for an additional surgical device to perform the maneuver. The implant proved to be effective in achieving the desired transposition in a progressive, gradual, and slow manner.

Ln64: This study aims to describe the RTP (Rampanelli Transposition Plate) implant developed by the authors in collaboration with the company Engevet (Engevet Ltd, Paraná, Brazil) and to evaluate the TTT technique using the developed implant. We hypothesize that the implant can facilitate tibial tuberosity transposition in a slow, progressive, and gradual manner.

Ln193: The outcomes of this ex vivo investigation substantiated the effectiveness of the RTP implant in executing the transposition maneuver in a slow, progressive, and gradual manner, thereby corroborating the hypothesis.

Ln302: The RTP implant has demonstrated efficacy in facilitating the transposition of the tibial tuberosity in canine cadavers, owing to its capability to gradually and steadily transpose the tuberosity without necessitating supplementary devices.

**Q: Considering that bone integrity after transposition was the primary outcome of the study, a postoperative CT assessment (CT was performed only in pre-surgical planning) could have had a greater sensitivity in detecting small tibial crest fissures.**

R: Thank you for the comment. We agree that postoperative CT assessment would provide greater sensitivity for detecting small fractures of the tibial crest. However, it was unfortunately not feasible to perform postoperative CT in this study. Therefore, the evaluation was carried out through visual inspection and radiographic imaging. Although radiography has lower sensitivity compared to CT, it remains an effective method for assessing fractures.

**Q: The described objective of evaluating complications is too generic and inconsistent with what was actually evaluated in the study and may lead the reader to think that this technique does not foresee complications in an absolute sense. It is recommended to be more accurate in describing the objective of the study, the results, the discussion and the conclusions making them consistent with what was actually evaluated.**

R: We thank the Editor for the positive feedback. We agree that, as previously written - particularly after considering the suggestions made by you and the Reviewers - the methodology described in this manuscript could lead readers to assume that no complications are associated with the use of the implant. Aware of the study's limitations, we revised portions of the objectives, results, discussion, and conclusion sections accordingly.

The sections of the manuscript that addressed complications not assessed in this study have been removed. Examples of sections that contained this information and were consequently adjusted are cited below. As this was a common observation from the Editor and Reviewers 1 and 2, all changes made in response to this point are detailed in the respective individual responses to them.

The sections of the manuscript that addressed complications not assessed in this study have been removed. Examples of sections that contained this information and were consequently adjusted are cited below. As this was a common observation from the Editor and Reviewers 1 and 2, all changes made in response to this point are detailed in the respective individual responses to them. Furthermore, to align with the changes made, the study's objective has also been altered.

Ln64: This study aims to describe the RTP (Rampanelli Transposition Plate) implant developed by the authors in collaboration with the company Engevet (Engevet Ltda, Paraná, Brazil) and to evaluate the TTT technique using the developed implant. We hypothesize that the implant can facilitate tibial tuberosity transposition in a slow, progressive, and gradual manner.

=====================

**Journal requirements:**

**When submitting your revision, we need you to address these additional requirements.**

**1. Please ensure that your manuscript meets PLOS ONE's style requirements, including those for file naming. The PLOS ONE style templates can be found at**

R: We have reviewed the manuscript and made all necessary adjustments to ensure it complies with PLOS ONE's style requirements, including proper file naming. We have used the PLOS ONE style templates provided in the link mentioned.

**2. To comply with PLOS ONE submissions requirements, in your Methods section, please provide additional information regarding the experiments involving animals and ensure you have included details on (1) methods of sacrifice, (2) methods of anesthesia and/or analgesia, and (3) efforts to alleviate suffering.**

R: Thank you for raising these points. Below are the requested revisions to the Methods section, in accordance with PLOS ONE submission requirements. Additionally, information concerning the ethics committee is detailed on line 71.

(1) Ln 74: The animals died from causes unrelated to this study and were sourced from the Pathology Service of the Federal University of Paraná – Palotina Sector.

**3. Thank you for stating the following financial disclosure:**

**[Engevet partially funded the research, which included the manufacturing of the implant and the instruments used].**

**At this time, please address the following queries:**

**a) Please clarify the sources of funding (financial or material support) for your study. List the grants or organizations that supported your study, including funding received from your institution.**

R: Engevet partially funded the research, exclusively by manufacturing the implant. This study was financed in part by the *Coordenação de Aperfeiçoamento de Pessoal de Nível Superior - Brasil (CAPES)* – Finance Code  001. Author GR received a research fellowship from CAPES, which supported the development of this study.

**b) State what role the funders took in the study.**

R: The funders had no role in study design, data collection and analysis, decision to publish, or preparation of the manuscript.

**c) If any authors received a salary from any of your funders, please state which authors and which funders.**

R: None of the authors received any salary or financial compensation from the sponsors of this study.

**Please include your amended statements within your cover letter; we will change the online submission form on your behalf.**

R: The information regarding the sources of funding has been duly included in the cover letter.

=====================

**Response to Reviewers**

**Reviewer #1:**

**Q: Lines 63-64: The statement does not seem correct to me, I believe that some of the implants described have the ability to dislocate the tibial tuberosity.**

**R:** We thank the reviewer for his/her suggestion. However, we consider that, to perform the transposition maneuver, the implants require the use of an auxiliary device or surgical instrument to displace the tibial tuberosity, such as the *Tibial Tuberosity Transposition Tool*. Although it is technically possible to use the pin itself as a lever, this approach is not recommended by the authors due to the potential risk of complications, including tibial crest fracture, and the lack of scientific publications reporting or supporting this technique. In the case of implants such as the *Rapid Luxation System*, a specific tool is available to perform the transposition. In this context, it is understood that the currently available implants do not inherently provide the means for tuberosity displacement, but only for its fixation. We acknowledge that the statement could be perceived as presumptuous; therefore, we have revised its wording to mitigate this impression.

Ln 61: …and require an additional device or surgical instrument to perform the transposition.

**Q: Line 73-74: The approval of the ethics committee is fine but the criteria for choosing bone explants, the conditions of donors, etc., should be described**.

R: We appreciate your valuable observation regarding the criteria for selecting bone explants and the conditions of the donors. We have included information regarding the age and weight of the animals and clarified that the specimens were obtained from the Pathology Service. After explantation, the bones were evaluated for signs of fractures, neoplasia, and open growth plates, as described “The inclusion criteria were bones without signs of fractures, bone callus, neoplasms, or open growth plates (lines 79-80).

Ln 72: Twenty-one tibias from adult dogs, aged between 1 and 12 years and weighing between 4 and 25 kg of small to medium-sized breeds, regardless of sex, were selected. The animals died from causes unrelated to this study and were sourced from the Pathology Service of the Federal University of Paraná – Palotina Sector.

**Q: Line 117: I probably didn't understand something well but I have a question: why was it chosen to indicate 2/4 and not the simplification of the fraction (divisione) that is ordinarily used (1/2)?**

R: Thank you for pointing out this detail. In other referenced studies, the value of two-quarters was used. However, we have decided to follow your suggestion and change it to one-half, as it is the standard and more commonly used notation. We have also made the corresponding adjustment in the Results section to ensure consistency with the proposed change.

Ln 122: …the transposition distance was standardized at one-half of the width of the tibial tuberosity, measured at the level of the patellar tendon insertion in the frontal plane.

Ln 173: Transposition required to achieve an equivalent to one-half of the width of the tibial tuberosity varied…

**Q: Lines 119-125: which software was the pre-operative planning performed with?**

R: Thank you for this important observation. We have added the information that the surgical planning was performed using the vPOP Pro software.

Ln 124: …within the vPOP Pro (VETSOS Education Ltd. – Shrewsbury, United Kingdom) software …

**Q: Line 143: it would be interesting to indicate to readers in terms of the rpm of the screw in order to comply with the speed previously reported in the literature.**

R: We thank the reviewer for the observation, have rewritten the sentence, and added information regarding the RPM. We consider that the revolutions per minute refer to the pitch of the transposition screw, and therefore, we have omitted this information from the text.

Ln 157: This process was executed slowly and gradually, at a rate of 0.7 mm/min, accompanied by a rotational speed of 1.4 revolutions per minute (RPM) for the M3x0.5 transposition screw and 1 RPM for the M4x0.7 screw [17,18].

**Q: Lines 149-150: did you also assess other parameters on the postoperative radiographs or only the presence or absence of fissuring/fracture of the tibial tuberosity?**

R: Thank you for the question. We also evaluated the implant placement and screw positioning radiographically. We rewrote the sentence structure to make it clearer, as the previous version did not properly convey that these assessments were performed both visually and radiographically. Additionally, another reviewer suggested including the specifications of the equipment used for performing the radiographic examinations.

Ln163: Upon completion of the implant application, the samples underwent visual and tactile inspection, as well as radiographic evaluation (Diafix Ltd, 500mA/125 kV - São Paulo, Brazil) in both mediolateral and craniocaudal projections. This was conducted to verify the displacement of the tibial tuberosity, identify any potential fractures of the tibial tuberosity, and assess the positioning of the implant and screws.

**Q: Lines 160-161: My main concern is about complications. I do not think it is correct to exclude complications due to implant failure or deformation without performing mechanical tests.**

R: We thank the reviewer for the comment. We share the same concern regarding potential complications. As mentioned, it is not possible to rule out implant failure or structural deformation without conducting specific mechanical testing. Considering this, we revised the presentation of the results to ensure that the conclusions are limited to what can be reliably stated based on the *in situ* assessment of the samples. We also revised the objectives, results, discussion, and conclusion sections to ensure consistency and alignment with the modifications made throughout the manuscript.

Ln175: All tibias from cadavers reached the stipulated transposition in a slow, gradual, and controlled manner. Furthermore, appropriate positioning of the transposition screw was observed near the insertion of the patellar ligament.

Ln179: In all specimens, the plate showed proper placement on the proximal tibial shaft, with sufficient bone stock to accommodate three fixation screws. Moreover, all screws remained well positioned. All specimens underwent visual and tactile inspection after the intended displacement, and no mobility suggestive of fracture was detected.

**Q: Lines 168-169: what should be the indications for implant failure? how can pull-out occur on a fully skeletonised tibia without a load test?**

R: We thank the reviewer for the suggestion and correction. We apologize for the mistake. We agree with your statement — it was a well-noted observation. Without mechanical load testing, it is not possible to detect implant failure or screw pull-out, as no force is applied to the system. Considering these aspects, we revised the sentence to clearly state what could actually be evaluated.

Ln186: Additionally, proper positioning of the screws and plate was observed, as well as the displacement of the tibial tuberosity.

**Q: Line 177: “The osteotomy line was shifted caudally”. This statement does not seem to me to be reflected in the attached images, wouldn't it be better to insert an image where this can be seen?**

R: We thank the reviewer for the correction. We were referring to the amount of remaining bone stock of the tibial tuberosity, which, when compared to early reports (ex: DeAngelis, 1971), shows a greater amount of available bone stock. We revised the sentence to more clearly convey the intended message to the reader. Furthermore, we have made an addition to the text (at the end of the paragraph in question) to enhance the clarity of the pertinent information.

Ln199: The incomplete osteotomy retains an intact segment of the crest distally, which imparts sufficient resistance to quadriceps traction during ambulation [17].

Ln203: Considering this information, the transposition screw proved effective in facilitating the displacement of the tibial crest.

**Q: Lines 240-243: how was the weakening of the tibial crest verified?**

R: We thank the reviewer for the question. To assess the integrity of the tibial crest, we performed a visual and tactile inspection. This consisted of applying manual pressure to the tibial crest; the occurrence of a fracture, detected by abnormal mobility or crepitus, would be considered a sign of tibial crest weakness. Additionally, post-implantation radiographs were taken to identify fractures or fissures in the tibial crest, which also characterize structural compromise (weakness).

We are aware that a biomechanical study would represent the ideal method for the unequivocal detection of tibial crest weakness. With this in mind, and to ensure that the description of our findings more accurately reflects what was directly observed, we have revised the terminology in the manuscript. The term 'weakness' has been replaced by 'fracture' in contexts where fracture is the primary indicator resulting from our analysis. The corresponding sentences, including in the Results section, have been duly modified.

Ln181: All specimens underwent visual and tactile inspection after the intended displacement, and no mobility suggestive of fracture was detected.

Ln240: In our findings, although preliminary due to the ex vivo nature of the study, no fractures were detected by visual and tactile inspection or radiographic assessment. In our findings, although preliminary due to the ex vivo nature of the study, no fractures were detected by visual and tactile inspection or radiographic assessment. The osteotomy performed in this study preserved residual bone stock and resulted in a safe osteotomy that effectively counteracted tensile distraction forces [17]. These initial results are encouraging and suggest that this approach may contribute to reducing complication rates related to tibial crest fragility in future clinical applications, consistent with the finding of Sullivan et al [17].

**Q: Lines 257-260: how has implant failure been ruled out?**

R: We thank the reviewer for the question. Firstly, we would like to apologize for the error. We acknowledge that, as this is a static evaluation (performed after transposition and without load application), it is not pertinent to assess 'implant failure' in this context. Therefore, we have accepted the comment, and the sentence has been changed to focus on the 'assessment of fractures'. Furthermore, in response to another reviewer's suggestion to condense this and the preceding paragraphs, the structure of the current paragraph has been modified. Consequently, the discussion regarding 'implant failure' is no longer detailed.

Ln240: In our findings, although preliminary due to the ex vivo nature of the study, no fractures were detected by visual and tactile inspection or radiographic assessment.

**Q: Figure 6: there are only five captions but a total of six figures.**

R: We apologize for the error. We have added the caption for Figure 6.

Ln330: **Figure 6.** (A) Radiograph in craniocaudal projection after tibial tuberosity transposition using the RTP implant. (B) Radiograph in mediolateral projection after TTT using the RTP implant.

**Reviewer #2:**

**Q: This is generally a well written article, but unfortunately requires some areas of revision to make it more suitable for publication. The text is well supported by pertinent references, and most, if not all, key references appear to have been included. It is an implant that could have an interesting future, but does need further studies (and I hope the authors progress to biomechanical studies).**

R: We thank the reviewer for the suggestions and pertinent comments. We also express our gratitude for your assessment of the implant as promising. The primary objective of this initial research phase was to demonstrate the capability of an implant to perform slow and gradual transposition of the tibial tuberosity without the need for auxiliary external devices. Furthermore, we would like to highlight that the next stage of our investigation will consist of performing a biomechanical study of this implant.

**Q:** **Language and grammar is generally good, but there are some areas where more appropriate words / phrases should be used, and there are some areas where I suspect language differences and / or autocorrect have created unfortunate errors – easily rectified! Details are given below.**

R: The manuscript has been revised according to the reviewer's recommendations. The changes made to the indicated sentences are demonstrated directly in the responses to each point. Additionally, presented below are some excerpts that, although not criticized, were corrected by the authors for general improvement of the text.

Ln87: Utilizing Autodesk Fusion 360 (Autodesk Inc., San Francisco, USA), a custom inverted ‘L’-shaped locking plate has been developed.

Ln91: The configuration of the plate enables the secure attachment of the implant to the tibial shaft, with one designated hole strategically located on the tibial tuberosity to enhance the transposition process.

Ln103: Subsequent to the design process, both the plate and fixation screw were manufactured from titanium (ASTM F163), while the transposition screw was constructed from 316L stainless steel.

Ln107: The osteotomy guide was meticulously engineered utilizing CAD Fusion 360 software (Autodesk Inc., San Francisco, USA) (Fig. 2A and 2B). It incorporates both a proximal and a distal aperture designed for 0.8 mm Kirschner wires, facilitating temporary fixation to the tibia. Additionally, it features a 1.2 mm high slot designated for blade insertion at the osteotomy site, effectively preventing potential displacement during the procedure. Four distinct sizes were developed, with varying slot lengths of 15, 25, 35, and 45 mm.

Ln124: In the medial view of the surgical plan within the vPOP Pro (VETSOS Education Ltd. – Shrewsbury, United Kingdom) software, the tibial mechanical axis was initially delineated. Subsequently, a perpendicular line to the mechanical axis was established at the insertion of the patellar tendon, and an additional line was drawn from the distal limit of the tibial crest to the caudal cortex, which was also perpendicular to the mechanical axis.

Ln153: To achieve this, the proximal Locking Screw (P) was initially inserted, succeeded by the distal Locking Screw (D), and lastly, the central Locking Screw (C) (Fig. 4B).

Ln160: The displacement was verified using a caliper, and the transposition time was recorded with a stopwatch.

Ln192: This study presents findings on the application of the newly developed RTP implant for TTT in tibias sourced from canine cadavers.

Ln196: The technique employed an incomplete osteotomy, aimed at preserving a bony bridge in the distal region and preventing proximal avulsion of the crest, while the implant mitigates the medial deviation of the newly transposed bone fragment.

Ln201: Additionally, the broader bone area affords enhanced resistance to the bending forces exerted during tibial tuberosity displacement, which may necessitate a greater resultant force to transpose the osteotomized segment.

Ln224: No bone fissures were detected during the radiographic evaluation conducted in this study. According to Filiquist et al., the manifestation of bone fissures in postoperative radiographs elevates the probability of fracture by 25%, as the formation of the fissure permits the quadriceps to exert traction on the tibial tuberosity, consequently subjecting the remaining cortex to cyclic loading, which may result in fractures [18].

Ln281: … in bone size…

Ln311: **Figure 1.** RTP implant developed for the tibial tuberosity transposition technique. (A) Three-dimensional visualization following the rendering of the implant. (B) Precision-machined implant for tibial tuberosity transposition.

Ln318: **Figure 2.** Osteotomy guide developed for executing the osteotomy during the tibial tuberosity transposition procedure. (A) Three-dimensional visualization following guide rendering. (B) Osteotomy guide subsequent to production.

**However, there are a few issues that need addressing (more details in body of text):**

**Q: - the article is perhaps a bit too long – it could (and should) be abbreviated**

R: We thank the reviewer for the suggestion. Your comments have been duly considered, and the text has been condensed in the sections where such an adjustment was deemed pertinent and feasible.

**Q: - there are some areas of superfluous information and a tendency to over-reference**

R: We thank the reviewer for the guidance. The superfluous information has been removed from the manuscript.

**Q: - there are some areas that need further detail (eg on translation screws).**

R: We thank the reviewer for the suggestion. In response to the guidance from the editor and reviewers, additional details have been incorporated into the manuscript. We would like to highlight that this supplementation of information focused, principally, on the features of the transposition screw.

**Q: - perhaps most significantly, the authors have made some assumptions / leaps in conclusion that are perhaps a bit optimistic, and make some unfair and/or inaccurate comparisons.**

R: We thank the reviewer for the suggestion. Throughout the manuscript revision process, we have adopted greater caution in our conclusions and comparisons, particularly concerning complications, in recognition of the inherent biomechanical limitation of the study.

**Q: In short, this is a paper that should ultimately be published, but with some new information added, some excess material removed, and the other areas lightly revised and appropriate rewording performed**.

R: We thank the reviewer for the positive feedback. In response, all suggested changes have been implemented aiming to improve the manuscript and to enable its publication.

**Cover:**
**Q: 15-16 were all authors equal contributors? Do we need two lines to state these? Perhaps it is a journal specific requirement - check PONE requirements.**

R: We thank the reviewer for the question. Regarding the author contribution statement, we inform that the authors were categorized into groups. Within each of these groups, the contributions of the respective members are considered equivalent. Although this approach may seem somewhat complex, it complies with a specific requirement of the journal PLOS ONE.

**Abstract:**
**Q: 19-21: First sentence – word order is odd (eg tibias in canines cadaers should be after TT) and is it realy investigating potential complications?**

R: Thank you for pointing out this detail. We have modified the sentence structure to improve clarity and adjusted the text to more accurately reflect the objective of the study.

Ln19: The present study aims to describe and evaluate the effectiveness and potential applicability of a new implant designed to transpose the tibial tuberosity in 21 cadaveric canine tibias.

**Q: 24: suggest add in calculated desired transposition Osteotomy was performed.**

R: We appreciate your suggestion and have revised the text accordingly.

Ln24: …calculated desired transposition. Subsequently, an osteotomy was performed…

**Q: 26-28: sentence needs rewording. Specifically the 21 tibias from canine cadavers. Should be earlier in the paragraph to make it read better.**

Thank you for the suggestion. The information has been duly included in the main document.

Ln19: The present study aims to describe and evaluate the effectiveness and potential applicability of a new implant designed to transpose the tibial tuberosity in 21 cadaveric canine tibias.

Ln26: After the osteotomy, the implant was fixed to the tibia, and the tibial tuberosity was transposed slowly and gradually using the transposition screw

**Intro:**

**Q: 40-41: – suggest change animals to dogs and delete ref to the stifle.. Add in origin after traumatic.**

R: Thank you for pointing out this detail.

Ln40: Medial patellar luxation is a common orthopedic condition in dogs, and it can be either congenital or traumatic origin.

**Q: 52: what do you mean by a fluid and dynamic process?**

R: Thank you for raising this question. We were referring to the fact that the assessment of patellar luxation cases is relative, and that the techniques employed must correspond to the deformities present in each case. In accordance with your suggestion, this paragraph and the subsequent one have been condensed and abbreviated and we have also restructured the sentence.

Ln51: The TTT is an effective technique employed for realigning the quadriceps extensor mechanism and can be combined with other procedures [9, 10].

**Q: 54: if its easy to perform, why the paper?**

R: We thank the reviewer for the question. The Tibial Tuberosity Transposition (TTT) procedure is widely used for the correction of patellar luxation and is often described as easy to perform; however, the associated complication rate can be considerably high. This manuscript aims to present a novel implant capable of performing the transposition simply and safely during the procedure, given that one of the common complications is tuberosity fracture due to rapid or careless displacement of the bone fragment. Furthermore, upon reflection, we considered that the term "easy" is not the most appropriate characterization for this procedure, and consequently, this designation has been removed from the manuscript.

Ln51: The TTT is an effective technique employed for realigning the quadriceps extensor mechanism and can be combined with other procedures [9, 10].

**Q:56: reluxation, not relaxation.**

R: Thank you for the correction. We apologize for this mistake. As this paragraph was condensed, the specific line containing the error was removed.

**Q: 59: suggest upto 19.8% or 3-19.8 (which you have quoted elsewhere)**

R: Thank you for the correction.

Ln55: …in up to 19.8% of dogs…

**Q: 52-60: these paragraphs could probably be combined and abbreviated.**

R: We thank you for pointing this out. In response, the two paragraphs in question have been restructured and consolidated into a single paragraph.

Ln51: The TTT is an effective technique employed for realigning the quadriceps extensor mechanism and can be combined with other procedures [9, 10]. However, surgical complications arising from the correction of patellar luxation using TTT are reported in approximately 13% to 48% of cases [8, 11-15]. Among these, patellar reluxation stands out as a significant complication, occurring in up to 19.8% of dogs, in addition to other adverse events such as implant failure or migration and tibial tuberosity avulsion [11, 16].

**Q: 62: missing “the”before TT.**

R: Thank you for pointing out this detail; we have made the correction.

Ln58: …for fixation after the TTT…

**Q:64: do you mean to inherently displace the TT? Perhaps add ïn situ’after fixed**

R: Thank you for your question. We were referring to the implant's capability to perform the transposition. Currently available implants require an additional device or instrument to achieve the displacement, such as the Tibial Tuberosity Transposition Tool (TTTT), and only fixate after displacement. We accepted your suggestion, added "in situ" to the sentence, and restructured the previous sentence for better clarification.

Ln61: …and require an additional device or surgical instrument to perform the transposition.

Ln62: …an implant “*in situ*” fixed…

**Q: 65: consider ïs desriable”or somehting along those lines rather than considered an option**

R: Thank you for the suggestion. The sentence in question has been altered.

Ln63: …desirable.

**Study model:**

**Q: 75: how were they selected? Was there any selection for age / size / sex?**

R: Thank you for your question. We have added the information to the main document.

Ln72: Twenty-one tibias from adult dogs, aged between 1 and 12 years and weighing between 4 and 25 kg of small to medium-sized breeds, regardless of sex, were selected. The animals died from causes unrelated to this study and were sourced from the Pathology Service of the Federal University of Paraná – Palotina Sector.

**Q: 79: what are osteotomy errors? Were they incomplete tibias in some cases?**

R: Thank you for your question. Osteotomy errors refer to cases in which the osteotomy was not positioned as planned or when the extent of the cut exceeded the predetermined limits. All tibias used in the study were intact. We revised the original sentence to improve clarity.

Ln80: Bones with iatrogenic damage, such as osteotomy errors, were excluded.

**Q: 80: was 6deg for 12 hours sufficient for adequate defrosting?**

R: Thank you for your question. As only bones individually wrapped in gauze were used, the defrosting time was considered sufficient.

**Plate:**

**Q: 84: First sentence superfluous**

R: Thank you for pointing out this detail. We have removed the sentence.

**Q: 96: how does the screw engage in the plate and with the bone. Is the screw hole threaded? Was there anything to stop backing out. Were they selected to be just long enough to transpose the bone and then lock in. Were the screws blunt ended or tipped? Need more details. Not necessarily lengthy, but enough to allow the reader to (within reason) replicate**

R: Thank you for the question. The hole in the plate is threaded, as is the screw, allowing both components to engage and enabling the displacement of the tibial tuberosity through screw rotation. Backward movement of the screw is prevented by the thread engagement, ensuring stability during transposition. The screws are available in varying lengths (4 to 10 mm) to allow selection according to the required degree of transposition. However, in this study, only 10 mm screws were used, as it was an ex vivo model and any excess length following implantation did not present a problem. We also added a supplementary video to facilitate visualization of how the transposition screw achieves the displacement.

We revised the text to improve clarity and included details regarding the threaded hole in the paragraph describing the plate, as well as more specific information about the screw’s length, shape, and tip. We also added additional information to the caption of Figure 1B regarding the characteristics of the transposition screw.

Ln88: This plate is designed with three threaded holes intended for tibial fixation using locking screws, two additional holes for temporary fixation with Kirschner wires, and one threaded hole designated for the transposition screw.

Ln94: The transposition screw was developed as a headless and fully threaded screw with a flat tip to allow the gradual displacement of the osteotomized bone fragment, without interference from locking between the screw head and the plate surface, unlike conventional locking screws. These screws vary in length (4 to 10 mm), depending on the desired degree of transposition (Fig. 1B).

Ln313: Green arrow: Transposition screw—a headless screw with a flat tip and full-length threading, designed to engage with the threaded hole of the plate. The rotation of this screw facilitates controlled displacement of the tibial tuberosity in accordance with the desired correction

Ln433: **S1**. Accelerated video demonstrating tibial tuberosity transposition using the RTP implant.

**Q: 99: what do you mean by implant systems? Do you mean 5 different plate sizes. What about Left vs Right?**

R: Thank you for the question. Yes, we were referring to size, we apologize for the lack of clarity. We have added the information to the main document.

Ln101: In consideration of the extensive variety of sizes among canines, five implant sizes were recommended, accompanied by distinct variations for the right and left sides.

**Q: 114: what CT windowing was employed?**

R: Thank you for the question. We have added the information to the main document.

Ln118: The images were acquired in 0.6 mm slices and three-dimensionally reconstructed using a bone window (WL 287; WW 332) to perform the preoperative planning for TTT of each sample.

**Q: 117: one-half (rather than two quarters) would read better**

R: Thank you for pointing out this detail. In other referenced studies, the value of two-quarters was used. However, we have decided to follow your suggestion and change it to one-half, as it is the standard and more commonly used notation. We have also made the corresponding adjustment in the Results section to ensure consistency with the proposed change.

Ln122: …the transposition distance was standardized at one-half of the width of the tibial tuberosity, measured at the level of the patellar tendon insertion in the frontal plane.

Ln173: Transposition required to achieve an equivalent to one-half of the width of the tibial tuberosity varied…

**Q: 118: at what level was the TT width measured?**

R: Thank you for the question. We have added the information to the main document.

Ln122: …the transposition distance was standardized at one-half of the width of the tibial tuberosity, measured at the level of the patellar tendon insertion in the frontal plane.

**Q: 119: perhaps better to state what plane was utilized**

R: Thank you for the suggestion. We have added the information to the main document.

Ln123: …in the frontal plane.

**Implant application:**

**Q: 132: I presume it was a medial approach? Whilst relatively obvious, this is not made clear.**

R: Thank you for the question. As the bones were dissected, no surgical approach to the soft tissues was performed. However, we understand that your question extends beyond the surgical access itself. Therefore, we have included additional information in the manuscript regarding the medial implantation approach.

Ln143: The osteotomy guide was positioned on the medial aspect of the tibia using two Kirschner wires so that the osteotomy line aligned with Gerdy's tubercle, making the osteotomized tibial crest 30% of the tibial width in the sagittal plane (Fig. 4A).

Ln151: Following the osteotomy, the implant was secured to the medial aspect of the tibia, ensuring that the insertion site for the transposition screw was aligned with the patellar tendon insertion.

**Q: 133-134: was it aligned with or just near tubercle of Gerdy? It kind of contradicts what it is in the previous section.**

R: We appreciate your observation and apologize for the previous error. The term has been corrected to “aligned” in the earlier section to ensure consistency and clarity in the manuscript.

Ln129: To accurately identify the tibial crest and the osteotomy region, a red line was strategically drawn, aligned with Gerdy's tubercle and extending distally to bisect the tibia into 30% cranial and 70% caudal proportions, employing the white lines as reference points (Fig. 3).

**Q: 135-136: You do not state an osteotomy was made! How did you measure 80% and 60% while making the cut?**

R: We appreciate your correction and apologize for the mistake. We have included a statement indicating that the osteotomy was performed, and, at the end of the paragraph, we detailed how the osteotomy measurements were obtained.

Ln146: Subsequently, an incomplete osteotomy was performed.

Ln149: The osteotomy length for both cortices was measured using a Castroviejo caliper.

**Q: 142: “screw was inserted”...where? This sentence could also be made clearer. Eg “..and advanced until the premeasured amount of displacement was achieved”, or something like that.**

R: Thank you for the suggestion. We revised the sentence.

Ln155: Subsequently, the transposition screw was inserted into the designated threaded hole and advanced until the tibial tuberosity reached the pre-measured displacement as delineated in the surgical plan (Fig. 5A, 5B, 5C, 5D).

**Q: 143: how was the rate of advancement measured?**

R: We thank the reviewer for the question. The rate of advancement was measured using the rotation per minute (RPM) of the wrench turns. Since the transposition screw was designed with standardized thread pitches, it was possible to control the speed based on this information. We have rewritten the sentence and included details about the wrench turns, referring to them as rotation per minute (RPM).

Ln157: This process was executed slowly and gradually, at a rate of 0.7 mm/min, accompanied by a rotational speed of 1.4 revolutions per minute (RPM) for the M3x0.5 transposition screw and 1 RPM for the M4x0.7 screw [17,18].

**Q: 147: how were they assessed – visually? Xray? CT? In particular, how could you assess the integrity without mechanical testing?**

R: Thank you for the question. The assessments were performed both visually and radiographically. The visual evaluation involved visual and tactile inspection of the tibial crest at the end of the transposition. We also revised the corresponding sections to maintain consistency with the reviewers’ suggestions and ensure textual coherence. Additionally, we understand the reviewer’s concern regarding the absence of mechanical testing. However, in the cases of a fracture or instability of the tibial tuberosity, these conditions can also be identified radiographically.

Ln163: Upon completion of the implant application, the samples underwent visual and tactile inspection, as well as radiographic evaluation (Diafix Ltd, 500mA/125 kV - São Paulo, Brazil) in both mediolateral and craniocaudal projections. This was conducted to verify the displacement of the tibial tuberosity, identify any potential fractures of the tibial tuberosity, and assess the positioning of the implant and screws.

**Results:**

**Q: 152: how was the size selection made? Eyeball? Measured from the CT? Other?**

R: Thank you for the question. The implant size selection was performed by comparing the implant measurements, obtained from the technical drawing, with the measurements of the medial aspect of the tibia as assessed on computed tomography. This information has been added to the manuscript in the Material and Methods section.

Ln138: Implant size selection was based on its dimensions, obtained from technical drawings, and on measurements of the medial tibial aspect, made from tomographic images. These measurements were performed using vPOP Pro (VETSOS Education Ltd. – Shrewbury, United Kingdom) software.

**Q: 156: one-half, not 2-quarters**

R: Thank you for the correction. We have changed all values in main document.

Ln173: Transposition required to achieve an equivalent to one-half of the width of the tibial tuberosity varied…

**Q: 157: probably better to use period rather than comma in decimal figures**

R: Thank you for the correction. It was a typographical error, which has already been corrected.

Ln174: (mean: 5.7 ± 1.32 mm)

**Q: 162:** **Table 1 – why were all (bar one) >0.5, when this was your measured target? Not a problem in itself, but may warrant some comment in the text / discussion – eg was it a limitation of measurement, technical difficulty of applying the screw?**

R: We thank the reviewer for the question and suggestion. This is an excellent observation. To achieve the displacement rate of 0.7 mm/min, as indicated in the manuscript, we divided one full turn of the screwdriver into four parts (0.25 turn every 15 seconds). Using the M4×0.7 transposition screw as an example, one full turn results in a 0.7 mm advancement. Therefore, to achieve the desired displacement rate, one complete turn per minute was performed, split into 0.25 turn increments every 15 seconds.
The screw rotation was visually controlled, as commonly done during surgical procedures. Thus, minor variations in screw advancement may have occurred due to slight imprecision in manual handling.

**Q: 167: evaluation of what? Presumably of the operated limbs. What views were taken. Equipment?**

R: We thank the reviewer for the question and corrections. Yes, we were referring to the evaluation of the operated limbs. This information has been added to the manuscript, including the radiographic views taken and the equipment used. The specifications of the equipment used have been added to the Materials and Methods section, as the radiographic assessment is mentioned for the first time at this point in the manuscript.

Ln164: …as well as radiographic evaluation (Diafix Ltd, 500mA/125 kV - São Paulo, Brazil) in both mediolateral and craniocaudal projections.

Ln184: Radiographic evaluation in craniocaudal and mediolateral projections of the operated tibias showed no fissures or fractures associated with the TTT (Fig. 6).

**Discussion:
Q: 177: shifted caudally relative to what?**

R: We thank the reviewer for the correction. We were referring to the amount of remaining bone stock of the tibial tuberosity, which, when compared to early reports (ex: DeAngelis, 1971), shows a greater amount of available bone stock. We revised the sentence to more clearly convey the intended message to the reader. Furthermore, we have made an addition to the text (at the end of the paragraph in question) to enhance the clarity of the pertinent information

Ln199: The incomplete osteotomy retains an intact segment of the crest distally, which imparts sufficient resistance to quadriceps traction during ambulation [17].

Ln203: Considering this information, the transposition screw proved effective in facilitating the displacement of the tibial crest.

**Q: 184: it’s not really deformation per se – deformation is not a force**

R: We thank you for the correction and the insightful observation. We apologize for the mistake and have corrected the sentence in the manuscript.

Ln208: The deformation behavior under tension depends on the speed at which the force is applied…

**Q: 186: not bone adaptation – this has a specific meaning in bone biology sp preferable to use alternative wording**

R: We thank the reviewer for the correction and suggestion. We apologize for the use of the incorrect term. The sentence has been revised.

Ln209: …therefore, it is recommended that the transposition be performed slowly and continuously to prevent the occurrence of fractures, respecting the yield point and the elastic properties of the bone…

**Q: 205-243: this is too long, and contains more detail than really necessary, and so should, in my opinion, be shortened. You also tend to jump around a bit – would be preferable to change the order, grouping comments on say one pin vs 2 pin together. Also, be consistent in pin vs k-wire**

R: We thank the reviewer for the suggestion and correction. We have followed the guidance, condensed the text, and ensured consistency in the terminology used to refer to the pins.

Ln230: The literature describes various methods for direct fixation of the transposed TT, with variable complication rates reaching up to 37% in different pin configurations [22 - 25]. A concern regarding TT fixation is the potential for bone fragility induced by the insertion of implants into the tibial crest [17, 18, 23]. Conversely, the use of an adjacent cortical screw has shown a low complication rate [18]. In this sense, the transposition screw is proposed as an alternative to provide stability without requiring transfixing implants in the TT, as such implants can weaken this structure.

Ln237: In a study conducted by Rossanese et al. [12], it was reported that distal preservation of the TT is associated with reduced risk of complications. Aligned with this principle, the osteotomy technique employed in the present study maintains robust bone stock, which is crucial for resisting tensile forces [17]. In our findings, although preliminary due to the ex vivo nature of the study, no fractures were detected by visual and tactile inspection or radiographic assessment. The osteotomy performed in this study preserved residual bone stock and resulted in a safe osteotomy that effectively counteracted tensile distraction forces [17]. These initial results are encouraging and suggest that this approach may contribute to reducing complication rates related to tibial crest fragility in future clinical applications, consistent with the finding of Sullivan et al [17].

**Q: 224: in rather than on the TC?**

R: We thank the reviewer for the correction and apologize for the error. The sentence has been revised.

Ln232: A concern regarding TT fixation is the potential for bone fragility induced by the insertion of implants into the tibial crest [17, 18, 23].

**Q: 241-243: hard to prove there was no weakening You can state there was no fracture / fissure but cannot really state there was no weakening without further testing.**

R: We thank you for the correction. We agree that proving the absence of weakness is complex without additional tests. The sentence in question has been altered in the main document.

Ln240: In our findings, although preliminary due to the ex vivo nature of the study, no fractures were detected by visual and tactile inspection or radiographic assessment. The osteotomy performed in this study preserved residual bone stock and resulted in a safe osteotomy that effectively counteracted tensile distraction forces [17]. These initial results are encouraging and suggest that this approach may contribute to reducing complication rates related to tibial crest fragility in future clinical applications, consistent with the finding of Sullivan et al [17].

**Q: 244-248: this sentence has no ending – there is no meaning to it. Also, are you mentioning all 3 techniques in the paper? If so, you are missing one (paper is spacer pin vs complete osteotomy + 2 pins and TBW vs partial osteotomy and 2 pins).**

R: We thank the reviewer for the correction and the question. The sentence in question has been restructured for greater conciseness and clarity. In response to your inquiry, we confirm that our initial consideration involved three techniques. However, the sentence has been duly corrected to specify that the discussion refers to the two techniques compared in the cited study.

Ln248: In a study by Sullivan et al. [17], no statistical difference was observed in ultimate failure force and stiffness between three fixation techniques: (i) an incomplete osteotomy fixed with a spacer pin, (ii) two pins and Tension Band Wire (TBW) with complete osteotomy, and (iii) an incomplete osteotomy fixed with two pins into the tibial crest.

**Q: 253: you say “as a result...” (of the transposition being performed by the implant device) “...there were no complications” but you cannot reasonably conclude that there were no complications due to this difference.**

R: We thank the reviewer for their consideration. We have revised the sentence so that it focuses on the outcomes that can be effectively assessed within the scope of our study.

Ln252: Although a tensile test was not performed in our study, the transposition screw utilized is designed to prevent segment return, a function analogous to that of a spacer pin, which makes these initial findings promising. Furthermore, when a spacer pin is used, displacement of the tibial crest is performed with a dedicated device, the Tibial Tuberosity Transposition Tool (TTTT); in contrast, the RTP implant utilizes its own transposition screw to achieve this displacement, thereby eliminating the need for an additional device. This study did not include a biomechanical analysis; however, we obtained satisfactory results without any fractures occurring during the transposition process.

**Q: 257: what do you mean by bone consolidation?**

R: We apologize for the translation error.

Ln261: …bone healing…

**Q: 257-260: Its not really a fair comparison. As you rightly acknowledge, there is no biomech analysis. All you can conclude is that no immediate implant failure was noted when the implant was applied.**

R: We thank the reviewer for the observation. We apologize for the premature conclusion and agree that the comparison was not appropriate. Consequently, the sentence has been reformulated: the new approach establishes a correlation between complication rates resulting from failures and the importance of preliminary *ex vivo* studies, both for implant development and for mitigating failures in clinical contexts.

Ln261: The literature indicates that implant failure occurs before bone healing in 2.1% to 13.8% of cases, resulting in unsuccessful TTT [10,13,14,18]. Given these rates, the development of new implants is essential. *Ex vivo* studies serve as critical preliminary steps to assess implant functionality and adaptability, enabling early validation and supporting progression to biomechanical studies and clinical evaluations. Accordingly, the RTP implant showed satisfactory functional performance in this study, supporting its suitability for future biomechanical testing.

**Q: 261-266: be cautious in your comparisons here. Whilst plates are involved in both systems (yours and theirs), it is a different method of application, and especially given the lack of detail on your transposition screw and how it engages (or otherwise) with the bone – so cannot be directly compared. It is however worth mentioning the plating systems available (but try not to make it too lengthy).**

R: We thank the reviewer for the correction. In response, the text has been made more concise, and all suggested changes have been duly implemented.

Ln268: Literature reports describe the stabilization of TTT using plates, notably involving fixation of the plate into the tibial crest [11,25]. One example is the Rapid Luxation System (RLS) implant (Rita Leibinger Medical, Tuttlingen, Germany) [11], which employs a spacer secured with screws to the tibial crest to maintain the transposition.

**Q: 266-267: unfair comparison – theirs was a clinical caseload vs ex-vivo study. Hard to definitvely compare the lack of fractures in such different models.**

R: We thank the reviewer for the feedback. We agree that the comparison is inappropriate and that fracture behavior in *ex vivo* versus *in vivo* studies is difficult to compare. Consequently, we have revised the sentence to discuss both implants without making a direct comparison of their respective complications.

Ln272: In contrast, the RTP implant was developed to obviate the need for direct fixation to the tibial tuberosity. Its functionality relies on the transposition screw, which prevents the TT from returning to its original position, thereby simplifying the transposition process.

**Q: 269: typo: “does not require fixation”.**

R: We thank you for the correction. We have corrected the typographical error.

Ln272: In contrast, the RTP implant was developed to obviate the need for direct fixation to the tibial tuberosity**.**

**Q: 272: delete “for its use”.**

R: We thank you for the correction. The sentence in question has been altered in the manuscript.

Ln277: However, the tibial shaft must have sufficient bone stock to accommodate the plate [11].

**Q: 278-280: we observed...tuberosity. What do you mean to say with this? What is your comparison with xray measurements? Is there a possible reference for this?**

R: We thank the reviewer for their pertinent comment. After careful consideration, we acknowledge that the statement 'We observed that CT is important for accurately planning the measurement of the displacement in millimeters necessary to transpose the tuberosity' and the subsequent sentence regarding screw length planning, although reflecting the clinical utility of CT, could be interpreted as inferences not directly substantiated by specific comparative data presented in this manuscript. To ensure the focus remains on the core results of our research and to avoid any ambiguity, we have opted to remove the sentences. We believe that this change makes the manuscript more concise and directly aligned with the evidence presented and referenced. We appreciate your contribution to the improvement of our manuscript.

**Q: 282-289: a little long. Good to recognise limitations and potential areas to improve the device and research ideas, but this could be made shorter.**

R: Thank you for the suggestion and feedback. Following the suggestion, we reduced the size of the text and removed the information regarding the decrease in the number of holes, given that, without a biomechanical analysis, it is unfeasible to confirm that this alteration would be beneficial for the implant.

Ln291: The implant can be improved; for instance, its plate thickness could be reduced, as it is positioned in an area with minimal muscle coverage, which, hypothetically, could reduce the risk of implant exposure. However, these observations do not result from a mechanical and clinical analysis of the implant.

**Q: 290+: I think this paper merits some further discussion on limitations**

R: We thank the reviewer for the suggestion. We have followed the guidance and expanded the discussion on the limitations, considering both the specific aspects of the present study and those related to the implant.

Ln283: A biomechanical study is required to evaluate the implant, particularly about a tensile test designed to determine the failure mode of the transposition screw, the failure mode of the tibial crest, and the response of the construct to implant pull-out.

Ln295: It is important to emphasize that this ex vivo study demonstrates the implant's capability to perform the transposition in a slow and gradual manner. However, as this study did not include a biomechanical evaluation under loading conditions, it does not describe potential complications that could arise when loads are applied to the implant. Although the current results are promising, this study provides a foundation for future investigations into the implant's efficiency.

**Q: 293-294: I think I know what you are trying to say, but it reads as though the quads traction during both extension and flexion. Could be improved in wording, And I’m not sure stimulate is the most appropriate word here – perhaps induce?**

R: We thank the reviewer for the suggestion. The corresponding sentence has been altered in the main document."

Ln286: In live patients, quadriceps traction can induce implant failure and avulsion of the tibial tuberosity (avTT), corroborating the need for a clinical study regarding the developed implant.

**Q: 295: warrants biomech study prior to any clinical study please!**

R: We thank the reviewer for the comment. We would also like to inform you that the next stage of our investigation will involve the biomechanical evaluation of this implant.

**Figures:**

**Q: 319: typo in Ltd.**

R: Thank you for the correction. We have corrected the typographical error in the sentence.

Ln322: **Figure 3.** Planning of the Tibial Tuberosity Transposition Procedure in vPOP Pro (VETSOS Education Ltd. – Shrewbury, United Kingdom). Green line - tibial mechanical axis. White lines - orientation lines for locating the osteotomy site. Red line – tibial tuberosity osteotomy line.

**Q: Fig5 B: are you concerned that from a clinical perspective, if a transposition of >50% was required (in this particular imaged bone, or maybe less in others), advancing the transposition screw will actually engage the tibial shaft rather than the TT?**

R: We thank the reviewer for the question. Yes, we share this concern. In our study, we established a displacement limit of 50% for the tibial tuberosity, as a displacement exceeding this threshold would reduce bone contact and could lead to bone healing failure in clinical situations. In cases where excessive tibial tuberosity displacement is necessary, the Tibial Tuberosity Transposition (TTT) technique is considered unfeasible. Under such circumstances, the use of derotational tibial osteotomies, such as Proximal Tibial Osteotomy (PTO), is recommended. However, after this study, we refined the implant design. The screw was then angulated 5 degrees superiorly to decrease the angle formed between it and the osteotomy line. This modification aims to reduce the risk, even in applications of the Tibial Tuberosity Transposition (TTT) technique that approach or exceed its ideal indications.
