# Supplementary material for: Evaluation of a new implant for Tibial Tuberosity Transposition in dogs: An ex vivo study
Source: PLoS One. 2025 Nov 4;20(11):e0335274. doi: 10.1371/journal.pone.0335274 (PMC12585020; doi:10.1371/journal.pone.0335274)
Supplement: Attachment — Submitted filename: Response_to_Reviewers_auresp_2.docx [file pone.0335274.s004.docx]

Dear Academic Editor and Reviewers

RE: PONE-D-25-12917 “Evaluation of a New Implant For Tibial Tuberosity Transposition in Dogs: An Ex Vivo Study”

We would like to thank for the opportunity to submit a revised version of our manuscript, and we believe that the referees presented thoughtful and constructive comments on our manuscript, and we are comfortable in addressing each one in this revised version.

Please find below responses to each comment. Changes in the manuscript are highlighted in red in a ‘tracked’ file. The highlighted version and a clean version of the revised manuscript were uploaded to the PLOS One submission site. All authors have read and approved the revised version of the manuscript as submitted.

During the process of revising the manuscript in response to the suggestions from the Editor and the Reviewers, we identified additional points where the text could be improved for greater clarity and readability. We have taken the initiative to make these supplementary corrections, which are duly highlighted in the text. However, we would like to emphasize that should the Academic Editor and the Reviewers prefer the previous version, we are entirely willing to revert these modifications.

We hope that you find our responses satisfactory. Thank you for your consideration of our manuscript. If you need any additional information, please, do not hesitate to contact us.

Yours sincerely,

The Authors

**Evaluation of a New Implant for Tibial Tuberosity Transposition in Dogs: An Ex Vivo Study**

**Response to Reviewers**

Comments: During the process of revising the manuscript in response to the suggestions from the Editor and the Reviewers, we identified additional points where the text could be improved for greater clarity and readability. We have taken the initiative to make these supplementary corrections, which are duly highlighted in the text. However, we would like to emphasize that should the Academic Editor and the Reviewers prefer the previous version, we are entirely willing to revert these modifications.

Ln51: The TTT is an effective technique for realigning the quadriceps extensor mechanism and can be combined with other procedures [9, 10].

Ln87: A custom inverted 'L'-shaped locking plate was created using Autodesk Fusion 360 (Autodesk Inc., San Francisco, USA).

Ln90: The design allows for secure attachment of the implant to the tibial shaft, with one designated hole strategically positioned on the tibial tuberosity to improve the transposition process.

Ln101: Considering the wide range of sizes among dogs, five implant sizes were recommended, with distinct variations for the right and left sides. After the design process, both the plate and fixation screw were made from titanium (ASTM F163), while the transposition screw was made from 316L stainless steel.

Ln123: The tibial mechanical axis was initially delineated in the medial view of the surgical plan within the vPOP Pro (VETSOS Education Ltd. – Shrewsbury, United Kingdom) software.

Ln132: The tibial crest measurement determined values corresponding to 80% of the osteotomy line on the medial surface and 60% on the lateral surface.

Ln162: Upon completion of the implant application, the samples underwent visual and tactile inspection and radiographic evaluation…

Ln219: Surgical complications are common in cases of patellar luxation correction using TTT, with studies reporting rates of 10 to 40% [12,20-22]. The most common complication is patellar reluxation, with rates between 3% and 19.8% [8,14,21]. Other frequent issues include fractures or avulsion of the tibial tuberosity (TTAv), implant failure, and implant migration [8,10,12-14,20]. These are classified as major complications that require surgical revision for correction [8]. In this study, we did not observe fractures of the tibial crest or implant failure. However, since this was an *ex vivo* study using tibias from canine cadavers, other complications such as patellar reluxation, TTAv, and implant migration were not evaluated.

Ln246: These initial results are promising and indicate that this approach may help reduce complication rates related to tibial crest fragility in future clinical applications, aligning with the findings of Sullivan et al. [17].

Ln262: The literature shows that implant failure happens before bone healing in 2.1% to 13.8% of cases, leading to unsuccessful TTT [10,13,14,18]. Due to these rates, developing new implants is crucial. *Ex vivo* studies are important initial steps to test implant performance and adaptability, allowing early validation and helping move to biomechanical studies and clinical tests. Therefore, the RTP implant demonstrated good functional performance in this study, confirming its potential for future biomechanical testing.

**Reviewer #1:**

**I believe that the answers are satisfactory, only one doubt remains. The study is static as it is performed on anatomical pieces, but when the implant is mounted on patients who will use the limb, what element will be able to guarantee that the transposition screw maintains the level of depth chosen at the time of surgery and does not tend to change its position (unscrew), before healing, with motor activity?**

**If there are no blocking elements, this aspect should be hypothesized and indicated among the limits of this study**

R: We thank the reviewer for the positive feedback and the pertinent question. Indeed, during the conduction of the study, we also considered the possibility of screw loosening. To mitigate this potential complication, we are collaborating with the responsible design engineer on an optimization of the implant design. The main modification will consist of optimizing the thread profile geometry, aiming to increase friction and maximize resistance to loosening under axial load. We acknowledge the validity of this concern and, therefore, the possibility of screw loosening has been included as one of the limitations in the discussion of our study.

Ln272: Another limitation is the absence of a locking mechanism for the transposition screw, which could hypothetically lead to its loosening or back-out before bone healing in live patients. For this reason, the implant will be optimized to prevent such micromotion before bone healing, with a view to future clinical applications.

**Reviewer #2:**

**Thank you for your revision and addressing our comments and suggestions.
While the paper has been improved significantly, there are still some areas that require some (minor) revisions. Suggested changes are listed below. Most are just suggested minor word changes to help readability, but there are some areas of possible repetition that could be combined and condensed.**

R: We thank the reviewer for their positive and encouraging feedback. In accordance with your guidance, we have implemented all indicated corrections to improve the manuscript's readability. Sections with potential repetitions have likewise been adjusted and condensed. We greatly appreciate your acknowledgment that the text has significantly improved, and we hope that this revised version meets an even higher standard of quality. The specific changes are detailed in the point-by-point responses to each of your comments.

**L20. reads oddly - suggest delete the "in 21 cadaveric canine tibias". Could be included instead in L23.**

R: We thank you for the correction. The information in question has been duly added to the indicated sentence.

Ln22: Computed tomography was performed on 21 cadaveric canine tibias to plan the surgical technique and calculate the desired transposition.

**L24. Calculate.**

R: We thank you for the correction. The term in question has been duly corrected.

Ln23: … calculate…

**L24 suggest add in osteotomy "of the tibial tuberosity".**

R: We thank the reviewer for the suggestion. The term in question has been added to the corresponding sentence.

Ln24: …an osteotomy of the tibial tuberosity was performed…

**L41 can be of either...**

R: We thank you for the correction. The term in question has been duly corrected.

Ln40: …can be of either…

**L61 but what about digital / manual transposition.**

R: We thank the reviewer for the valuable question. Indeed, manual transposition of the tibial tuberosity is a viable approach, harkening back to the first TTT methods described in the literature. However, in recent years, a growing trend towards the use of specific devices to ensure greater procedural precision has been observed. The manual approach can result in lower precision due to micromovement inherent to the manipulation. For this reason, we have chosen to focus on describing implants and methods that represent ‘innovations’ in the technique, but we have adopted the recommendation, and the manuscript has been revised to include information on the manual transposition method.

L61: …additional device or manual pressure to perform the transposition.

**L62 don't need " "around in situ; perhaps 'applied' rather than fixed?**

R: We thank the reviewer for the suggestion. The corresponding sentence has been duly altered in the manuscript.

Ln62: …implant applied after…

**L85 suggest comprising rather than formed by.**

R: We thank the reviewer for the suggestion. The corresponding sentence has been duly altered in the manuscript.

Ln85: …comprising a plate for fixation…

**L107 suggest delete meticulously.**

R: We thank the reviewer for the suggestion. The corresponding sentence has been duly altered in the manuscript.

Ln106: ...was engineered utilizing…

**L130 suggest delete strategically.**

R: We thank the reviewer for the suggestion. The corresponding sentence has been duly altered in the manuscript.

Ln129: …was drawn…

**L143 suggest delete "After planning...began" (superfluous).**

R: We thank you for the suggestion. The sentence in question has been removed from the manuscript. As the sentence was deleted in its entirety, it is not possible to demonstrate this change visually, however, that the removal was made on Ln142

**L153 Labels missing from images supplied.**

R: We thank the reviewer for the correction. The missing caption for Figure 4 has been added, and the sentence in question has been corrected. Additionally, to improve clarity and visual correspondence, the labels in the figure have been changed from letters to symbols.

Ln152: To achieve this, the proximal locking screw was initially inserted, succeeded by the distal locking screw, and lastly, the central locking screw (Fig. 4B).

Ln318: **Figure 4.** (A) Positioning of the osteotomy guide with two Kirschner wires. (B) Fixation of the RTP implant at the height of the patellar tendon insertion with three locking screws, inserted in sequential order: proximal (arrowhead), distal (asterisk), and central (arrow).

**L179-181 Seems to be repeating content, unless you mean specifically about assessment post procedure - if so, would benefit from clearer wording**

R: We thank the reviewer for the guidance. Indeed, we acknowledge the presence of repetitive information in the text. Therefore, the sentence in question has been removed to eliminate the redundancy, as can be seen on Ln177.

**L198 mitigates not really appropriate here.**

R: We thank the reviewer for the correction. The term has been duly replaced with a more suitable alternative in the manuscript.

Ln193: …while the implant maintains the lateral deviation…

**L199-200 repetition of content re crest intact - could be condensed with earlier line on this.**

R: We thank the reviewer for the suggestion. In response, the sentence in question has been removed, and the paragraph has been restructured to eliminate repetition.

Ln192: The technique employed an incomplete osteotomy, aimed at preserving a bony bridge in the distal [17], while the implant maintains the medial deviation of the newly transposed bone fragment.

**L202 do you mean applied rather than resultant force?**

R: We thank the reviewer for this question. The reviewer is correct; we were indeed referring to the "applied force." The sentence has been revised.

Ln196: …a greater applied force…

**L227 suggest reword - the fissure doesn't permit the force - he force is being applied regardless**.

R: We thank the reviewer for the correction. The sentence in question has been rephrased.

Ln230: …this occurs because the quadriceps exert traction on the tibial tuberosity, subjecting the remaining cortex to cyclic loading, which may ultimately lead to fracture [18].

**L237-242 Again, repeats about the bone stock . width - suggest combine with earlier part of discussion.**

R: We thank the reviewer for the positive feedback. We would like to clarify the logical structure of these paragraphs in the 'Discussion' section. The paragraph in question focuses specifically on the potential of incomplete osteotomy to reduce complications, based on the findings of Rossanese et al. (2019) and Sullivan et al. (2024). In contrast, the preceding paragraph, although also mentioning incomplete osteotomy, discusses the transposition technique itself and the implant's capability to perform it.

We agree that there was some repetitiveness in the original wording. To address this, we have readjusted the sentences to eliminate redundancies and improve the transition, as also suggested in another point (Ln192). However, we respectfully disagree with the suggestion to combine the two paragraphs. We believe that merging them would impair the clarity of the manuscript by conflating two distinct topics (the technique and its complications), which would leave the argumentation about complications displaced from its logical context. Therefore, we have opted to keep the paragraphs separate but with improved wording to avoid repetition

Ln192: The technique employed an incomplete osteotomy, aimed at preserving a bony bridge in the distal region [17], while the implant maintains the lateral deviation of the newly transposed bone fragment.

Ln240: In a study conducted by Rossanese et al. [12], it was reported that distal preservation of the TT is linked to a lower risk of complications. Consistent with this principle, the osteotomy technique used in this study preserves strong bone stock, which is essential for resisting tensile forces [17]. Although our findings are preliminary due to the ex vivo nature of the study, no fractures were observed through visual and tactile inspection or radiographic assessment, and a solid bone bridge was maintained.

**L242-244. unfortunately this does not prove it counteracts tensile forces, as no mechanical studies have been performed.**

R: We thank the reviewer for the correction. Indeed, we acknowledge that the statement cannot be substantiated within the scope of this work, as no mechanical tests were performed. Therefore, the sentence in question has been readjusted.

Ln245: and a solid bone bridge was maintained.

**L268-279 Feels out of place. Perhaps move earlier in discussion, before discussing implant failure etc.**

R: We thank the reviewer for the suggestion. In response, the paragraphs in question have been repositioned in the text to precede the discussion of complications. As a result of repositioning the paragraphs in question, the order of the references in the text has been altered. Throughout the manuscript, the references affected by this change have been highlighted in red on their respective lines (L208, L220, L223, L235, L237).

Ln207: Literature reports describe the stabilization of TTT using plates, notably involving fixation of the plate into the tibial crest [11,19]. One example is the Rapid Luxation System (RLS) implant (Rita Leibinger Medical, Tuttlingen, Germany) [11], which employs a spacer secured with screws to the tibial crest to maintain the transposition. In contrast, the RTP implant was developed to obviate the need for direct fixation to the tibial tuberosity. Its functionality relies on the transposition screw, which prevents the TT from returning to its original position, thereby simplifying the transposition process.

Ln215: Plates for fixation after performing TTT have demonstrated satisfactory results in correcting tibial tuberosity deviation. However, the tibial shaft must have enough bone stock to support the plate [11]. In our study, all 21 tibias had sufficient bone stock for the placement of three locking screws to secure the plate.

Ln399:

1. Eskelinen EV, Suhonen AP, Virolainen JV, Liska WD. Tibial Tuberosity Transposition Fixation with a Locking Plate during Medial Patellar Luxation Surgery: An Ex Vivo Mechanical Study. *Vet Comp Orthop Traumatol*. 2022;35(2):96-104. doi:10.1055/s-0041-1736217
2. Arthurs GI, Langley-Hobbs SJ. Complications associated with corrective surgery for patellar luxation in 109 dogs. *Vet Surg*. 2006;35(6):559-566. doi:10.1111/j.1532-950X.2006.00189.x
3. Linney WR, Hammer DL, Shott S. Surgical treatment of medial patellar luxation without femoral trochlear groove deepening procedures in dogs: 91 cases (1998-2009). *J Am Vet Med Assoc*. 2011;238(9):1168-1172. doi:10.2460/javma.238.9.1168
4. Wangdee C, Theyse LF, Techakumphu M, Soontornvipart K, Hazewinkel HA. Evaluation of surgical treatment of medial patellar luxation in Pomeranian dogs. *Vet Comp Orthop Traumatol*. 2013;26(6):435-439. doi:10.3415/VCOT-12-11-0138
5. Zide AN, Jones SC, Litsky AS, Kieves NR. A Cadaveric Evaluation of Pin and Tension Band Configuration Strength for Tibial Tuberosity Osteotomy Fixation. *Vet Comp Orthop Traumatol*. 2020;33(1):9-14. doi:10.1055/s-0039-1693968
6. Stanke NJ, Stephenson N, Hayashi K. Retrospective risk factor assessment for complication following tibial tuberosity transposition in 137 canine stifles with medial patellar luxation. *Can Vet J*. 2014;55(4):349-356.
7. Birks RR, Kowaleski MP. Combined Tibial Plateau Levelling Osteotomy and Tibial Tuberosity Transposition: An Ex Vivo Mechanical Study. *Vet Comp Orthop Traumatol*. 2018;31(2):124-130. doi:10.1055/s-0038-1625973

**L284 reword. "about a tensile test" doesn't really read correctly. Perhaps delete "about...designed".**

R: We thank the reviewer for the suggestion. We acknowledge that the original sentence was confusing and contained excessive information. Accordingly, the sentence has been restructured to improve its clarity.

Ln276: A biomechanical study is required to evaluate the implant, particularly to determine the failure mode of the implant and the tibial crest.

**L287 avTT - different acronym used elsewhere for this (L218).**

R: We thank the reviewer for the correction. The acronym has been duly corrected in the manuscript.

Ln278: …TTAv…

**L297 repetition of the biomech evaluation.**

R: We thank the reviewer for the correction. To avoid repetition, the sentence regarding the biomechanical evaluation has been removed from the preceding paragraph (Ln 286).

**Fig 4 - need letters added in (L153).**

R: We thank the reviewer for the correction. The missing information has been duly added to the caption in question.

Ln318: **Figure 4.** (A) Positioning of the osteotomy guide with two Kirschner wires. (B) Fixation of the RTP implant at the height of the patellar tendon insertion with three locking screws, inserted in sequential order: proximal (arrowhead), distal (asterisk), and central (arrow).
